# In a Hospital Ward

**Published:** 1886-10-23

**Authors:** 


### IN A HOSPITAL WARD.

By a Nurse.

'Twas midnight, and the lights were burning low,
And all around was only suffering;
Then thinking on the sadness, wasted lives,
Hearts broken, crippled limbs, and buried hopes
Of others and myself, I moaned,
"Has God forgotten?"

And near me lay one who was passing swift
Away from us, from all the toil and pain,
His brow was moistened with the chill of death,
And on his face an awful look of fear,
And gazing on his features, once again I moaned,
"Has God forgotten?"

Then as I lay and murmured, all at once
My eyes were opened, so that I could see
A presence, in the dimly lighted ward,
Passing from bed to bed all silently,
Leaving behind a wondrous peace and calm,
And whispered words of love.

And lo! beside the dying one He stayed,
And bending low He laid his hand on him,
A pierced hand; and when I saw that scar,
And all the wondrous beauty of His face,
I knew the Lord stood there.

And then the vision faded, and we seemed
Once more alone, but now I knew
Who came and stood beside us, though unseen,
Soothing the lonely hearts with words of love,
And turning to the dying one, I saw,
He smiled and slept.

